# High expression of RUVBL1 and HNRNPU is associated with poor overall survival in stage I and II non-small cell lung cancer patients

**DOI:** 10.1007/s12672-022-00568-0

**Published:** 2022-10-15

**Authors:** Justyna Durślewicz, Jakub Jóźwicki, Anna Klimaszewska-Wiśniewska, Aleksandra Zielińska, Paulina Antosik, Dariusz Grzanka, Marcin Braun

**Affiliations:** 1 Department of Clinical Pathomorphology, Faculty of Medicine, Collegium Medicum in Bydgoszcz, Nicolaus Copernicus University, Torun, Poland; 2 Department of Pathology, Chair of Oncology, Medical University of Lodz, Lodz, Poland

**Keywords:** Non-small cell lung cancer, NSCLC, Prognostic biomarkers, Nuclear matrix protein, RUVBL1, HNRNPU

## Abstract

The present study aimed to investigate expression levels and prognostic significance of RUVBL1 and HNRNPU in stage I and II non–small-cell lung cancer (NSCLC) patients. Therefore, we evaluated immunohistochemical staining of RUVBL1 and HNRNPU, as well as RNA-seq data from public sources, and the results were evaluated concerning overall survival (OS) and clinicopathological features. We found that RUVBL1 and HNRNPU proteins and mRNA levels were higher in tumor tissues as compared to adjacent/normal tissues. RUVBL1 (p = 0.013) and HNRNPU (p = 0.021) high protein levels were independent prognostic factors for poor OS. Also, the multivariate analysis in the TCGA dataset revealed that high *RUVBL1* (p = 0.064) and *HNRNPU* (p = 0.181) mRNA levels were not significantly associated with prognosis. However, the co-expression status of these markers (R + H +) was independently associated with poor OS both in the TCGA dataset (p = 0.027) and in our cohort (p = 0.001). In conclusion, combined and individual expression of RUVBL1 and HNRNPU proteins, as well as R + H + mRNA status, may serve as potential prognostic biomarkers for NSCLC. This study adds to the previous observations that RUVBL1 and HNRNPU might be novel and promising therapeutic targets and markers for prognostic evaluation.

**Supplementary Information:**

The online version contains supplementary material available at 10.1007/s12672-022-00568-0.

## Introduction

Nuclear matrix proteins (NMPs) are part of the nuclear scaffold and perform essential functions like spatial DNA organization, regulation of transcription, and replication. There is also robust evidence that different NMPs are involved in pathologic processes such as tumorigenesis and tumour differentiation [1, 2]. Thus, it can be speculated that NMPs may serve as valuable markers of malignancies including prognostication in oncology, and new targets for molecular therapies [3].

RUVBL1 (Rvb1, pontin, TIP49), one of the NMPs, is a member of the ‘ATPases associated with diverse cellular activities’(AAA +) superfamily of proteins being involved in a wide variety of functions crucial to cell physiology. RUVBL1 possesses both ATPase and DNA helicase activity. As a part of numerous protein complexes, it has a significant role in the cell cycle, chromatin ATP-dependent remodelling, DNA repair, histone modification, and apoptosis [4–6]. By controlling C-RAF kinase’s phosphorylation, RUVBL1 influences the RAF/MEK/ERK pathway, which is known to be overactivated in several cancer types, including non-small lung carcinoma (NSCLC), leading to cancer progression [7].

Heterogeneous nuclear ribonucleoprotein U (HNRNPU), the nuclear scaffold attachment factor A (SAF-A), is DNA- and RNA-binding NMP. It controls various cell functions such as transcription, nuclear chromatin organization, alternative pre-mRNA splicing, telomere-length regulation, inhibition of apoptosis, angiogenesis, or cell invasion [8–10]. Mounting evidence suggests that HNRNP family members play an essential role in numerous cancers, including breast cancer, ovarian cancer, hepatocellular cancer, melanoma, bladder cancer, prostate cancer and pancreatic cancer [11–14].

Lung cancer remains the leading cause of cancer-related death worldwide, with NSCLC being the most prevalent histological type. Despite considerable improvement in NSCLC treatment and diagnostic options, a high percentage of NSCLC patients still have an unfavourable prognosis, even in the early stages of the disease [15–17].

To our knowledge, less than 30 articles have checked the significance of NMP in NSCLC, and only four studies focused on HNRNPU or RUVBL1 [18–21]. All of these studies provided mechanistic evidence of the biological importance of HNRNPU or RUVBL1 in NSCLC in vitro models; however, only one investigated possible associations with patients’ survival in tumoral material [19]. No study approached this issue in clinical material based on protein levels. Further examination of NMP within NSCLC would improve our knowledge of the molecular tumor landscape, which may lead to discovering new effective diagnostic methods, identifying new drug targets, and a better understanding resistance mechanisms to therapies. This seems even more attractive given that identification of RUVBL1 inhibitors is ongoing.

Combining in-house and publicly available data, our study aimed to explore the individual and combined prognostic value of the RUVBL1 and HNRNPU for stage I and II NSCLC patients.

## Materials and methods

### Patients and tissue specimens

The research was performed on formalin-fixed paraffin-embedded tissue (FFPE) specimens collected between 2010 to 2014 from 67 patients with NSCLC, who were diagnosed in the Franciszek Łukaszczyk Oncology Center in Bydgoszcz. The study material consisted of 67 samples of NSCLC tumors and 60 adjacent normal lung tissues. Patient clinicopathological characteristics are presented in Supplementary Table 1. A part of this cohort was included in our previous study [22]. The following variables were collected from the hospital files: gender, age, smoking history, grading, pathologic T stage (pT), pathologic N stage (pN), and staging. The tumors were reclassified according to the standardized TNM 8^th^ edition classification of the American Joint Committee on Cancer (AJCC) criteria.

The study was approved by the Ethics Committee of the Nicolaus Copernicus University in Toruń, Ludwik Rydygier Collegium Medicum in Bydgoszcz (no. KB 336/2018).

### Immunohistochemical reaction

Selected paraffin blocks were cut into 4-μm thick sections. The immunohistochemical (IHC) reactions were conducted using the BenchMark® ULTRA (Roche Diagnostics/Ventana Medical Systems, Tucson, AZ, USA) using anti-RUVBL1 rabbit polyclonal antibody (cat. no: HPA019948, Sigma-Aldrich, St. Louis, MO, USA) in a 1:400 dilution and anti-HNRNPU (cat. no: HPA058707, Sigma-Aldrich) in a 1:100 dilution. The visualization of the reactions was carried out using the ultraView Universal DAB Detection Kit (Roche Diagnostics/Ventana, Tucson, AZ, USA) according to the manufacturer's instructions and a previously approved protocol [22].

### Evaluation of immunohistochemical reaction

Two independent pathologists conducted the assessment using an Olympus BX53 (Olympus, Tokyo, Japan) light microscope at 20 × and 40 × original objective magnification. RUVBL1 and HNRNPU levels were quantified according to a modified H-score. The scoring system was established by adding the fraction of stained cells (FSC) multiplied by the percentage of cells multiplied by the intensity of each type. The intensity of staining was defined as 0-negative, 1-low staining, 2-moderate staining, and 3-strong staining. FSC was calculated using the number of stained cells per 1000 cells of the same type. The final staining score, ranging from 0 to 300, was categorized into two expression groups based on a specific discrimination cut-off set by the Evaluate Cutpoints software [23]. The cut-off point values for high and low expression of RUVBL and HNRNPU were as follows: < 100; ≥ 100, < 95; ≥ 95, respectively. We established a combination of the expression of the studied factors. RUVBL1^high^HNRNPU^high ^(R^+^H^+^) cases were analysed in comparison with the opposite expression pattern—RUVBL1^low^HNRNPU^low ^(R^−^H^−^).

### In silico analysis of TCGA data

The survival and gene expression data for The Cancer Genome Atlas (TCGA) cohort of 761 NSCLC patients were obtained from www.cBioPortal.org and UCSC Xena Browser (http://xena.ucsc.edu/). Stage I and II cases of NSCLC were included, which comprised 64.56% and 35.44% of the cohort, respectively. The data were divided into low-level and high-level expression groups according to the cut-off points provided by the Evaluate Cutpoints software. The cut-off point values for high and low expression of *RUVBL1* and *HNRNPU* were as follows: < 12.12; ≥ 12.12, < 14.77; ≥ 14.77, respectively. We created the expression combination of the factors examined. Cases with high co-expression of both *RUVBL1* and *HNRNPU*–*RUVBL1*^high^*HNRNPU*^high ^(*R*^+^*H*^+^) were analysed against the opposite expression pattern—*RUVBL1*^low^*HNRNPU*^low^(*R*^−^*H*^−^).

### Statistical analysis

Statistical analyses were performed using SPSS software packages version 26.0 (IBM Corporation, Armonk, NY, USA) and GraphPad Prism version 8.0 (GraphPad Software, San Diego, CA, USA). The Shapiro–Wilk test was applied to assess the normality of distribution. Due to non-normal distribution, the Mann–Whitney U-test was used to compare continuous variables. Categorical variables were compared using the Fisher’s exact test or the Chi-squared test. Survival curves were assessed using the Kaplan–Meier method, and differences were estimated based on the log-rank test. Data on disease duration were censored at the last time points, *i.e.* date of death from any cause or date of the last follow-up. The median follow-up was calculated using the reverse Kaplan–Meier estimator. Univariate and multivariate survival analyses were performed using the Cox proportional hazard regression model. The hazard ratios (HRs) and 95% confidence intervals (95% CIs) were also calculated. The results were considered statistically significant with p-value < 0.05.

## Results

### Patient characteristics of the study cohort

The overall median age at diagnosis was 62 years (ranging from 46 to 82 years), with 21 (31.34%) female and 46 (68.66%) male patients. In terms of histology, most of the patients had SCC, ADC, and LCC—36 (53.73%), 26 (38.81%), and 5 (7.46%), respectively. Among the 67 NSCLC patients, 64 (95.52%) were former or current smokers. There were 19 (28.36%) cases of moderately differentiated tumors (intermediate grade, G2) and 48 (71.64%) cases of poorly differentiated (high grade, G3). Regarding the pathologic T stage, there were 17 (25.37%), 25 (37.31%), and 25 (37.31%) cases ranging from pT3, pT2 to pT1, respectively. Sixty-one (91.04%) patients were characterized by a negative and 6 (8.96%) by a positive lymph node status. Twenty-two (32.84%) patients were stage IA, 14 (20.90%) patients were stage IB, 8 (11.94%) patients were stage IIA and 23 (34.33%) patients were stage IIB. Postoperative survival data were available for all the patients. The median follow-up time was 1990 (95%CI 1966–2014) days, and 70.15% (n = 47) of the patients died during follow-up, whereas 29.85% (n = 20) were still alive. The clinicopathological characteristics of the patients are summarized in Supplementary Table 1.

### Patient characteristics of the TCGA cohort

The overall median age of the patients at diagnosis was 68 years (ranging from 33 to 88 years), with 307 (40.34%) female and 454 (59.66%) male patients. Regarding histology, 374 (49.15%) patients had ADC, and 387 (50.85%) had SCC. There were 249 (32.72%) cases of pT1, 446 (58.61%) cases of pT2, and 66 (8.67%) cases of pT3. Of 761 patients, 171 (22.47%) had lymph node metastases, while 590 (77.53%) were N0. Stage I and II comprised 488 (64.13%), and 273 (35.87%), respectively. The median follow-up time was 913 (95%CI 826–1000) days, and 36% (n = 274) of the patients died during follow-up, whereas 64% (n = 487) were still alive. The clinicopathological characteristics of the patients are summarized in Supplementary Table 2. A comparison of the clinicopathological features of our cohort and the TCGA cohort is presented in Supplementary Table 3. The cohorts differed significantly in terms of pT and pN statuses (p < 0.001 and p = 0.008, respectively), but did not differ in terms of TNM stage (p = 0.112).

### Association between RUVBL1 protein and mRNA expression levels with clinicopathological characteristics of NSCLC patients

Expression of the investigated proteins in our cohort was evaluated with IHC in 67 NSCLC and 60 non-tumor adjacent tissues. IHC staining of RUVBL1 was detected in the nuclear, membrane and cytoplasmic compartments of NSCLC cells. We found no association of nuclear or membrane RUVBL1 expression with overall survival (OS) and clinicopathological features (Supplementary Fig. 1, Supplementary Table 4); therefore, our results comprised the cytoplasmic pattern only.

**Fig. 1:**
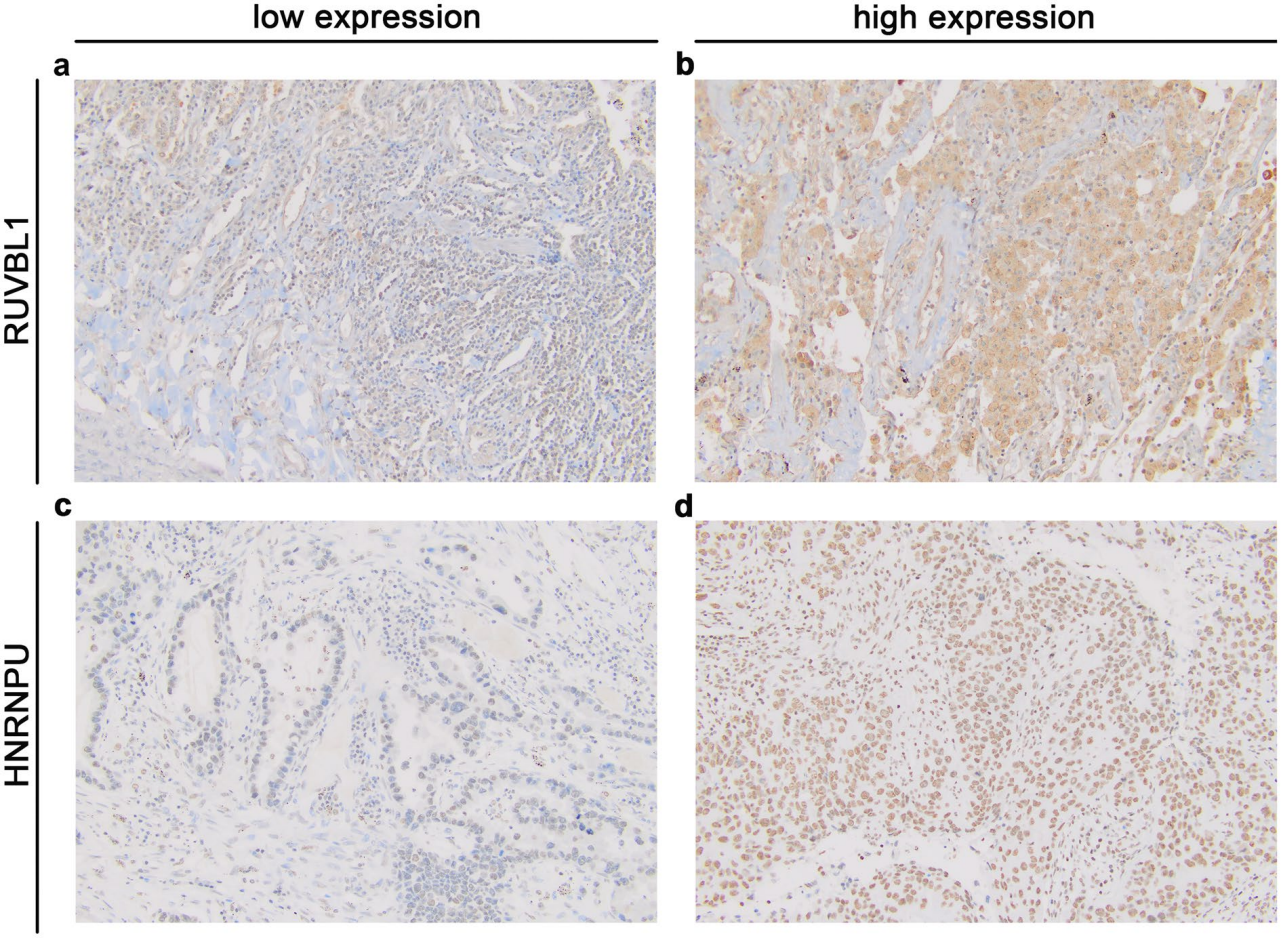
Representative immunohistochemical staining for RUVBL1 (**a**, **b**) and HNRNPU (**c**, **d**) in NSCLC tissues. **a** Low cytoplasmic expression of RUVBL1, **b** High cytoplasmic staining for RUVBL1, **c** Low nuclear expression of HNRNPU, **d** High nuclear staining for HNRNPU. Primary magnification × 20

**Fig. 2:**
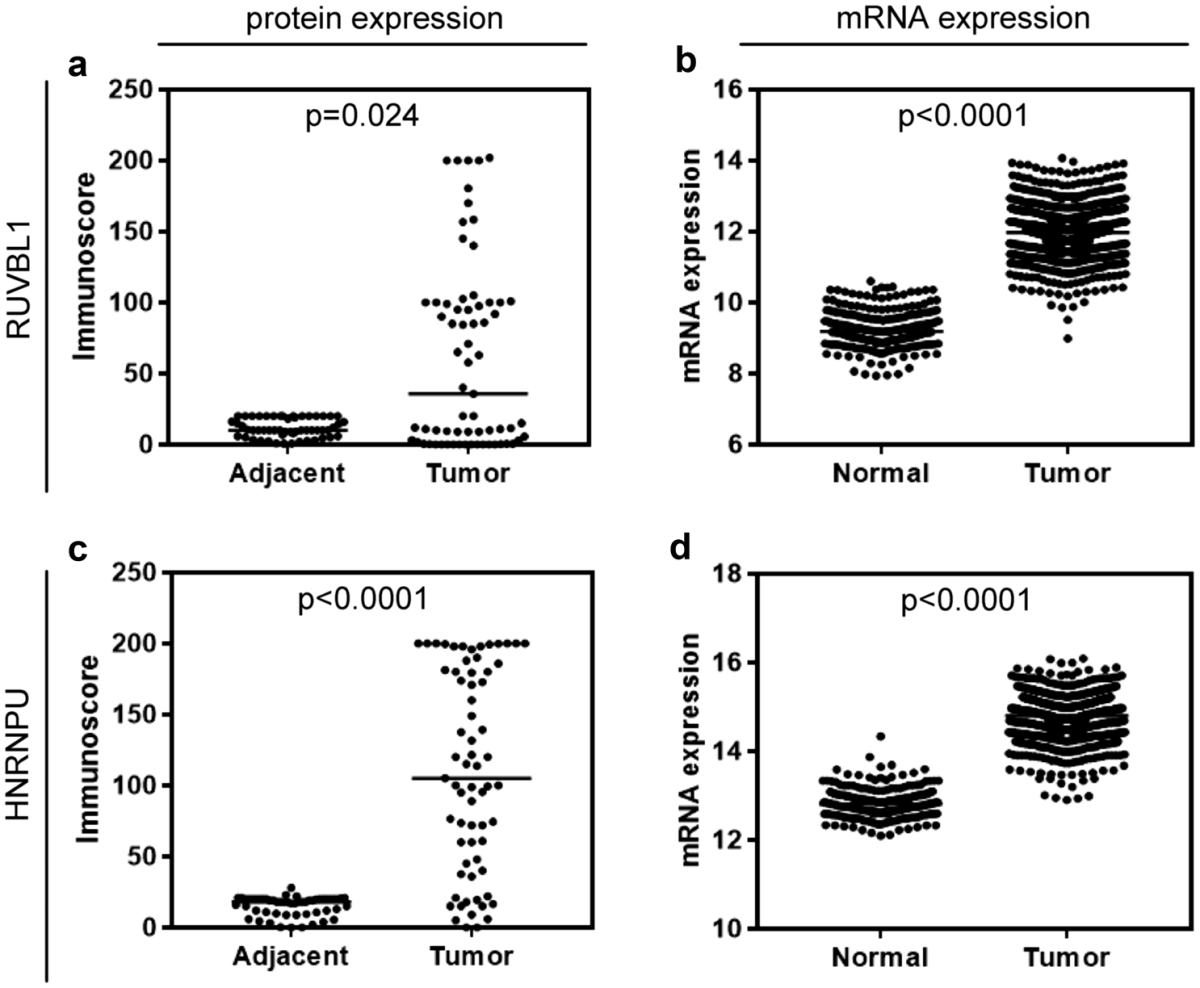
Protein and mRNA expression of *RUVBL1* and *HNRNPU* in tumor and adjacent tissues in NSCLC. **a** RUVBL1 protein expression levels in NSCLC tumors compared to noncancerous adjacent tissues; **b** *RUVBL1* mRNA expression levels in NSCLC tumors compared to normal tissues; **c** HNRNPU protein expression levels in NSCLC tumors compared to noncancerous adjacent tissues; **d** *HNRNPU* mRNA expression levels in NSCLC tumors compared to normal tissues

According to the established cut-off points, high cytoplasmic immunoreactivity of RUVBL1 was found in 14 (20.9%) NSCLC cases, whereas the remaining 53 (79.1%) demonstrated low or no expression. Representative images illustrating low and high IHC expression of RUVBL1 are presented in Fig. 1a–b. As shown in Fig. 2, RUVBL1 expression was increased in NSCLC tissues as compared to normal lung tissues (p = 0.024; Fig. 2a). Immunoreactivity of RUVBL1 was associated with histological type (p < 0.001). Positive expression of RUVBL1 was observed more frequently in the ADC type (n = 11; 42.31%) than in SCC (n = 2; 5.56%) and LCC (n = 1; 20.00%). No other relationships between RUVBL1 protein expression and clinicopathologic characteristics were found (Table 1).

**Table 1:** Association of RUVBL1 and HNRNPU, and clinicopathological features in our cohort of NSCLC patients (n = 67)

	Cases n (%)	RUVBL1			HNRNPU		
		+	-	P-value	+	-	P-value
		n = 14	n = 53		n = 39	n = 28	
Histological type							
ADC	26 (38.81)	11 (42.31)	15 (57.69)	**0.0021**	19 (73.08)	7 (26.92)	0.1252
SCC	36 (53.73)	2 (5.56)	34 (94.44)		17 (47.22)	19 (52.78)	
LCC	5 (7.46)	1 (20.00)	4 (80.00)		3 (60.00)	2 (40.00)	
Gender							
Females	21 (31.34)	6 (28.57)	15 (71.43)	0.3403	12 (57.14)	9 (42.86)	> 0.9999
Males	46 (68.66)	8 (17.39)	38 (82.61)		27 (58.70)	19 (41.30)	
Age							
< 62	30 (44.78)	5 (16.67)	25 (83.33)	0.5516	17 (56.67)	13 (43.33)	> 0.9999
≥ 62	37 (55.22)	9 (24.32)	28 (75.68)		22 (59.46)	15 (40.54)	
Smoking							
Never	3 (4.48)	1 (33.33)	2 (66.67)	0.5110	3 (100.00)	0 (0.00)	0.2592
Former/current	64 (95.52)	13 (20.31)	51 (79.69)		36 (56.25)	28 (43.75)	
Histologicgrade							
G2	19 (28.36)	7 (36.84)	12 (63.16)	0.0916	12 (63.16)	7 (36.84)	0.7843
G3	48 (71.64)	7 (14.58)	41 (85.42)		27 (56.25)	21 (43.75)	
pT status							
T1	25 (37.31)	6 (24.00)	19 (76.00)	0.3520	12 (48.00)	13 (52.00)	0.3572
T2	25 (37.31)	3 (12.00)	22 (88.00)		17 (68.00)	8 (32.00)	
T3	17 (25.37)	5 (29.41)	12 (70.59)		10 (58.82)	7 (41.18)	
pN status							
N0	61 (91.04)	13 (21.31)	48 (78.69)	> 0.9999	36 (59.02)	25 (40.98)	0.6885
N1	6 (8.96)	1 (16.67)	5 (83.33)		3 (50.00)	3 (50.00)	
Stage							
I	36 (53.73)	7 (19.44)	29 (80.56)	0.7723	22 (61.11)	14 (38.89)	0.6283
II	31 (46.27)	7 (22.58)	24 (77.42)		17 (54.84)	14 (45.16)	

*ADC *adenocarcinoma, *SCC *squamous cell carcinoma, *LCC *large cell carcinoma, *pT status* extent of the primary tumor, *pN status* absence or presence and extent of regional lymph node metastasis. Bold font indicates statistical significance

In turn, based on the cut-off values determined for *RUVBL1* mRNA, high expression was found in 324 (42.58%) NSCLC cases, while low in 437 (57.42%). In the TCGA cohort analysis, similarly to RUVBL1 protein expression, mRNA levels were significantly up-regulated in tumor tissues compared to non-cancer normal tissues (p < 0.001; Fig. 2b). Moreover, we observed significantly higher mRNA expression of *RUVBL1 *in males (n = 228; 50.55%) than in females (n = 96, 31.27%; p < 0.001). We did not find any associations between *RUVBL1* mRNA expression and age, pT, pN, and TNM stage (Table 2).

**Table 2:** Association of *RUVBL* and *HNRNPU,* and clinicopathological features in the TCGA cohort of NSCLC patients (n = 761)

	Cases	RUVBL1			HNRNPU		
		+	−	P-value	+	−	P-value
	n (%)	n = 324	n = 437		n = 398	n = 363	
Gender							
Females	307 (40.34)	96 (31.27)	211 (68.73)	** < 0.0001**	172 (56.03)	135 (43.97)	0.1036
Males	454 (59.66)	228 (50.22)	226 (49.78)		226 (49.78)	228 (50.22)	
Age							
< 68	374 (49.15)	156 (41.71)	218 (58.29)	0.6603	185 (49.47)	189 (50.53)	0.1280
≥ 68	387(50.85)	168 (43.41)	219 (56.59)		213 (55.04)	174 (44.96)	
pT status							
T1	249 (32.72)	98 (39.36)	151 (60.64)	0.4446	137 (55.02)	112 (44.98)	0.2981
T2	446 (58.61)	196 (43.95)	250 (56.05)		223 (50.00)	223 (50.00)	
T3	66 (8.67)	30 (45.45)	36 (54.55)		38 (57.58)	28 (42.42)	
pN status							
N0	590 (77.53)	250 (42.37)	340 (57.63)	0.8608	308 (52.20)	282 (47.80)	0.9309
N1	171 (22.47)	74 (43.27)	97 (56.73)		90 (52.63)	81 (4737)	
Stage							
I	488 (64.13)	198 (40.57)	290 (59.43)	0.1466	251 (51.43)	237 (48.57)	0.5454
II	273 (35.87)	126 (46.15)	147 (53.83)		147 (53.85)	126 (46.15)	

*pT status* extent of the primary tumor, *pN status* absence or presence and extent of regional lymph node metastasis. Bold font indicates statistical significance

### Association between HNRNPU protein and mRNA expression levels with clinicopathological characteristics of NSCLC patients

IHC staining of HNRNPU was detected in the nuclear compartments of NSCLC cells. Representative photographs showing expression of HNRNPU in NSCLC are presented in Fig. 1c, d. Thirty-nine samples (58.21%) of tumor tissue were characterized by low and 28 (41.79%) with high HNRNPU expression. We found that the expression of HNRNPU in our cohort was significantly upregulated as compared with non-tumor adjacent tissues (p < 0.001, Fig. 2c). No relationships were observed between clinicopathologic features and HNRNPU protein expression (Table 1). In the TCGA cohort, *HNRNPU* mRNA overexpression was observed in 398 (52.3%) cases. Furthermore, high expression of mRNA *HNRNPU* in NSCLC was also significantly up-regulated as compared to normal lung tissues (p < 0.0001; Fig. 2d). Correspondingly to protein analysis, no association was found between clinicopathological features and *HNRNPU* mRNA expression (Table 2).

### Survival outcomes based on protein and mRNA expression levels of *RUVBL1* in NSCLC patients

**Fig. 3:**
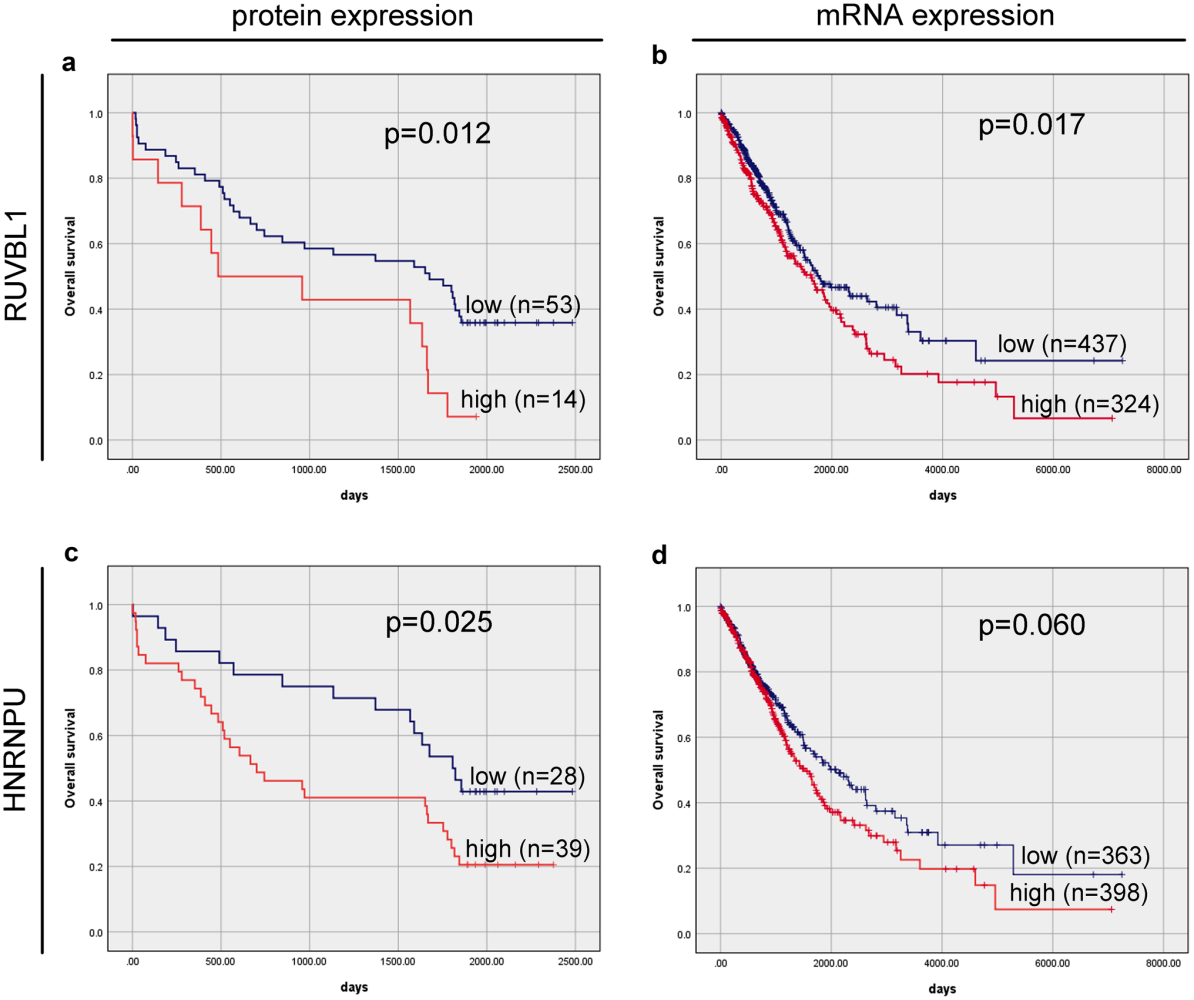
Kaplan–Meier survival curves and log-rank test for overall survival of NSCLC patients based on **a** RUVBL1 protein expression and **b** *RUVBL1* mRNA expression, **c** HNRNPU protein expression and** d** *HNRNPU* mRNA expression. n – number of patients in the group

The Kaplan–Meier survival curves revealed that OS was significantly worse in NSCLC patients with high vs. low RUVBL1 protein expression (median OS of 484 and 1676 days, respectively; HR = 2.25, 95%CI 1.17–4.32; p = 0.015; Table 3; Fig. 3a). In the multivariate analysis, following adjustment for gender, age, and TNM stage RUVBL1 high protein expression was described as an independent poor prognostic factor for OS (HR = 2.32, 95% CI 1.19–4.53; p = 0.013, Table 3). To verify the above results, the prognostic significance of *RUVBL1* mRNA levels in the TCGA cohort were examined. These analyses showed a significant association between *RUVBL1* mRNA expression and OS of NSCLC (p = 0.017; Fig. 3b). *RUVBL1* mRNA high vs. low expression was found to be related to a significantly shorter OS for NSCLC patients (median OS of 1778 vs.1632 days, respectively; HR = 1.33, 95%CI 1.05–1.69; p = 0.018; Table 4). The results were not significant in the multivariate Cox analysis adjusted for gender, age, and stage (HR = 1.26, 95%CI 0.99–1.60; p = 0.064; Table 4).

**Table 3:** Univariate and multivariate Cox proportional hazards models for OS of the NSCLC patients (n = 67)

Variable	Univariate analysis				Multivariate analysis: RUVBL1				Multivariate analysis: HNRNPU			
	HR	95% CI		P-value	HR	95.0% CI		P-value	HR	95.0% CI		P-value
RUVBL1	2.252	1.173	4.323	**0.015**	2.323	1.193	4.527	**0.013**	–	–	–	–
HNRNPU	1.973	1.075	3.620	**0.028**	–	–	–	–	2.070	1.118	3.831	**0.021**
Gender	1.116	0.609	2.044	0.722	1.106	0.599	2.040	0.748	1.041	0.563	1.923	0.899
Age	1.028	0.990	1.068	0.151	1.035	0.997	1.075	0.070	1.033	0.994	1.074	0.102
TNM stage	1.68	0.93	3.04	0.087	1.714	0.938	3.132	0.080	1.789	0.986	3.248	0.056
pT status	1.477	0.777	2.807	0.234	–	–	–	–	–	–	–	–
pN status	1.711	0.672	4.355	0.260	–	–	–	–	–	–	–	–

Bold font indicates statistical significance

**Table 4:** Univariate and multivariate Cox proportional hazards models for OS of the TCGA patients with NSCL (n = 761)

Variable	Univariate analysis				Multivariate analysis: RUVBL1				Multivariate analysis: HNRNPU			
	HR	95% CI		P-value	HR	95.0% CI		P-value	HR	95.0% CI		P-value
*RUVBL1*	1.332	1.051	1.689	**0.018**	1.257	0.987	1.603	0.064	–	–	–	–
*HNRNPU*	1.257	0.990	1.596	0.061	–	–	–	–	1.178	0.927	1.499	0.181
Gender	1.143	0.893	1.463	0.290	1.055	0.820	1.357	0.677	1.098	0.857	1.408	0.460
Age	1.017	1.004	1.031	**0.013**	1.018	1.005	1.033	**0.009**	1.018	1.004	1.032	**0.012**
Stage	1.589	1.248	2.024	** < 0.0001**	1.559	1.221	1.990	** < 0.0001**	1.574	1.233	2.008	** < 0.0001**
pT status	1.385	1.062	1.807	**0.016**	–	–	–	–	–	–	–	–
pN status	1.291	0.990	1.684	0.060	–	–	–	–	–	–	–	–

Bold font indicates statistical significance

### Survival outcomes based on protein and mRNA expression levels of *HNRNPU* in NSCLC patients

A Kaplan–Meier survival analysis indicated that NSCLC patients with high HNRNPU protein expression had lower OS rates than those with HNRNPU low expression level (OS median values of 701vs. 1807 days, respectively; HR = 1.97, 95%CI 1.08–3.62; p = 0.028; Table 3; Fig. 3c). This observation persisted as an independent prognostic factor for poorer OS in the multivariate Cox analysis after adjustment for age, gender, and stage (HR = 2.07, 95%CI 1.12–3.83;p = 0.021; Table 3). In the analysis of the TCGA data, NSCLC patients with high mRNA *HNRNPU* tended to survive for shorter periods than those with low expression (median OS of 1528 vs. 2086 days; HR = 1.26, 95% CI 0.99–1.60; p = 0.061; Table 4; Fig. 3d). In the multivariate analysis following adjustment for age, gender, and stage it was not an independent prognostic factor for OS (HR = 1.18, 95%CI 0.93–1.50; p = 0.181; Table 4).

### Overall survival analysis according to biomarker co-expression

**Fig. 4:**
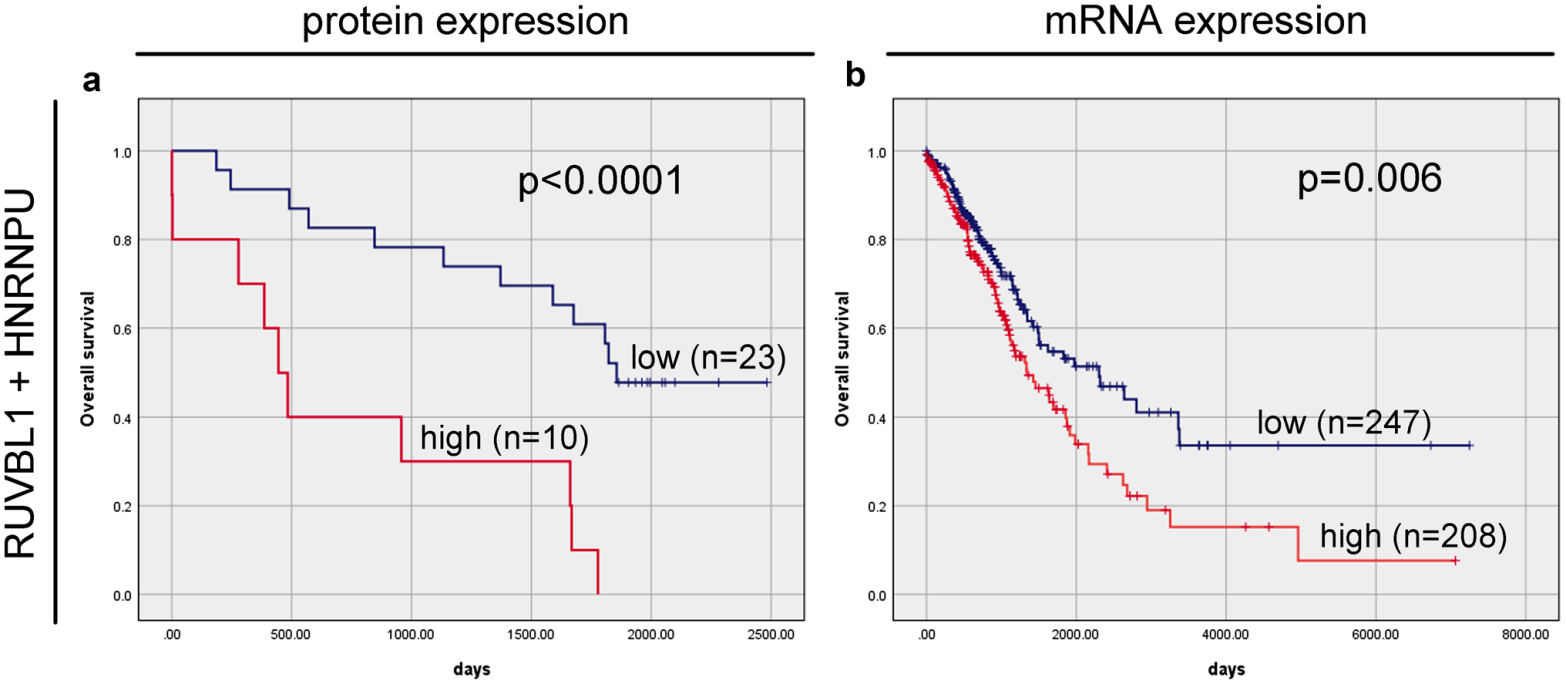
Overall survival analysis according to the combination of **A** RUVBL1 and HNRNPU proteins and **B** *RUVBL1* and *HNRNPU* mRNAs. *R*^*+*^*H*^*+*^ simultaneous high expression of RUVBL1 and HNRNPU proteins; *R*^*−*^*H*^*−*^ simultaneous low expression of RUVBL1 and HNRNPU proteins; *R*^+^*H*^+^ simultaneous high expression of *RUVBL1* and *HNRNPU* mRNAs; *R*^*−*^*H*^*−*^ simultaneous low expression of *RUVBL1* and *HNRNPU* mRNAs, *n* number of patients in the group

After evaluating the prognostic significance of RUVBL1 and HNRNPU protein and mRNA levels as individual markers in our cohort of NSCLC patients, their combined prognostic value was assessed. The Kaplan–Meier analysis revealed that the worst OS was observed in patients whose NSCLC co-expressed RUVBL1 and HNRNPU proteins at high levels (R^+^H^+^). In contrast, patients with the opposite expression profile of these proteins had a significantly longer OS (R^+^H^+^ vs. R^−^H^−^: 445 days vs. 1856 days; p < 0.0001; Fig. 4a). In unadjusted analysis, having R^+^H^+^ corresponds to a hazard ratio of 5.13 (95%CI 2.03–12.97) for the decrease in OS (p = 0.001; Table 5), and this effect was stable in the multivariate analysis adjusted for gender, age, and stage (HR = 5.427, 95%CI 2.08–14.18; p = 0.001; Table 5). The survival analysis of mRNA data from the TCGA cohort also revealed that combined *RUVBL1* and *HNRNPU* high expression (*R*^+^*H*^+^ mRNA vs. *R*^*−*^*H*^*−*^ mRNA, Fig. 4b) was significantly associated with poor OS (median OS of1338vs. 2304 days, respectively; HR = 1.550, 95%CI 1.13–2.12; p = 0.006; Table 6), and this finding persisted as significant in the multivariate analysis adjusted for gender, age and stage (HR = 1.437, 95%CI 1.04–1.98; p = 0.027; Table 6).

**Table 5:** Univariate and multivariate Cox proportional hazards models for combined expression of RUVBL1 and HNRNPU proteins (n = 33)

Variable	Univariate analysis				Multivariate analysis			
	HR	95% CI		P-value	HR	95.0% CI		P-value
R^+^H^+^	5.129	2.028	12.971	**0.001**	5.427	2.077	14.184	**0.001**
Gender	1.710	0.691	4.235	0.246	1.870	0.721	4.848	0.198
Age	1.039	0.980	1.102	0.202	1.072	1.005	1.144	**0.035**
TNM stage	1.517	0.651	3.532	0.334	1.899	0.748	4.819	0.177
pT status	1.284	0.522	3.160	0.586	–	–	–	–
pN status	1.622	0.477	5.521	0.439	–	–	–	–

*R*^*+*^*H*^*+*^ simultaneous high expression of RUVBL1 and HNRNPU proteins. Bold font indicates statistical significance

**Table 6:** Univariate and multivariate Cox proportional hazards models for combined expression of RUVBL1 and HNRNPU mRNAs (n = 455)

Variable	Univariate analysis				Multivariate analysis			
	HR	95% CI		P-value	HR	95.0% CI		P-value
*R*^+^*H*^+^	1.550	1.134	2.119	**0.006**	1.437	1.042	1.980	**0.027**
Gender	1.124	0.816	1.548	0.474	1.007	0.727	1.395	0.965
Age	1.014	0.996	1.032	0.123	1.014	0.996	1.032	0.128
Stage	1.481	1.080	2.030	**0.015**	1.409	1.021	1.945	**0.037**
pT status	1.389	0.976	1.977	0.068	–	–	–	–
pN status	1.253	0.895	1.755	0.189	–	–	–	–

*R*^+^*H*^+^ simultaneous high expression of *RUVBL1* and *HNRNPU* mRNAs. Bold font indicates statistical significance

## Discussion

In our study, we used the in-house cohort of stage I-II NSCLCs and demonstrated that RUVBL1 and HNRNPU protein expression levels were significantly higher in NSCLC tissues than in control tissues and associated with worse survival of patients. We then validated these results in the independent, publicly available dataset of stage I-II NSCLCs derived from the TCGA database.

Several studies have been conducted to evaluate the expression level and role of RUVBL1 in different human tumors [18, 20, 24–28]. The presented results indirectly corroborate that RUVBL1 may play an essential role in carcinogenesis, as we found that both protein and mRNA expression levels of *RUVBL1* were significantly higher in NSCLC samples than those measured in control tissues, which was also reported by Yenerall et al.[20] and Yuan et al.[18]. Furthermore, by array CGH followed by validation with qPCR, Dehan et al. demonstrated that *RUVBL1* was located within the amplified region of 3q21 and overexpressed in NSCLCs [29]. In agreement with our studies, high expression of RUVBL1 was also shown in a variety of human solid tumors, such as colorectal [26], hilar cholangiocarcinoma [27], renal cell carcinoma [24], and hepatocellular carcinoma [28]. Therefore, we next asked whether RUVBL1 protein expression was associated with available clinicopathological factors, including histological type, gender, age, grade, pT, pN and TNM stage. A statistically significant association was observed between RUVBL1 protein expression level and histological type, with RUVBL1 positivity rate occurring more frequently in ADC (42.31%) than SCC (5.56%), p = 0.002, which was also supported by Yenerall et al.[20]. RUVBL1 staining differences between the histological subtypes of NSCLC may result from completely different underlying mechanisms driving the development of these tumors. Previous studies from other authors have reported that high RUVBL1 expression was significantly associated with the aggressive clinical features of cancer [24, 27], but we did not observe any significant relationship. However, our study was limited by the number of subjects and the uneven distribution of the clinicopathological data. Furthermore, the patients in our cohort had stage I or II disease; therefore, we could not compare them with more advanced clinical stages.

The unadjusted survival analyses showed that stage I-II NSCLC patients with high RUVBL1 protein expression are characterised by shorter OS than those with low RUVBL1 expression. Similarly to high RUVBL1 protein levels, high *RUVBL1* mRNA levels were associated with a poorer OS. To identify RUVBL1 as a potential independent risk factor, the level of RUVBL1 expression and clinicopathological characteristics were evaluated by the multivariate Cox regression analysis. We found that RUVBL1 protein expression was as independent prognostic factor, which remained stable in the multivariate analysis adjusted for gender, age, and stage for predicting the survival of NSCLC patients. Similar results of Kaplan–Meier analysis were provided by Yenerall et al. even though they analysed RUVBL1 protein expression by three groups (low, medium, or high)[20], while in our considerations, expression was categorized into low and high levels according to the optimal cut-off point. Furthermore, Yenerall et al. analysed nuclear-cytoplasmic expression of RUVBL1. In our study, we observed cytoplasmic, nuclear, and membrane staining of the RUVBL1 protein in the cancer cells; however, the Kaplan–Meier survival and clinicopathological features analyses found no significant association of nuclear and membrane expression with NSCLC OS; therefore, our results included the cytoplasmic staining pattern only. Previously, findings comparable to ours were published by Zhang et al., who showed that high expression of RUVBL1 in cytoplasm positively correlated with unfavourable outcomes in renal cell carcinoma patients [24]. In the cited study, nuclear RUVBL1 expression was not significantly correlated with patients’ survival, which is in line with our results [24]. This corresponds with in vitro experiments demonstrating that RUVBL1 was predominantly expressed in the cytoplasm of renal cell carcinoma cell lines [24] and pancreatic ductal adenocarcinoma cell lines [30]. These reports suggest the presence of cytoplasm-specific RUVLB1 functions beyond its role in chromatin remodelling, DNA damage response, and transcriptional regulation in the nucleus. However, the detailed molecular mechanism of this process is still elusive.

Based on previous studies and our results, it may be proposed that upregulated RUVBL1 expression is associated with an adverse prognosis in NSCLC. To support this theory, Yuan et al. showed that RUVBL1 knockdown could effectively inhibit the proliferation of ADC lung cell lines [18]. Moreover, Yenerall et al. demonstrated that RUVBL1/2 inhibition enhances the efficacy of radiation in cancerous cells but not in normal ones, which appears to be a beneficial property in future preclinical development [20]. Undoubtedly, these findings indicate a potentially important role of RUVBL1 in the biology and clinical behaviour of NSCLC.

HNRNPU is NMP that has been reported to play a critical role in the pathogenic process of numerous malignancies. However, its function and clinical significance in NSCLC remain unclear. In our investigation, the mRNA and protein expression levels of HNRNPU were significantly higher in NSCLC samples than in non-cancerous adjacent tissues. Contrary to our evidence, Pan et al. demonstrated downregulation of *HNRNPU* in various NSCLC cell lines [21]. Li et al. also showed that mRNA expression levels of *HNRNPU* detected by qRT-PCR were reduced in LSCC tissues compared to paratumoral tissues [31]. However, in our investigation, as the first group, we evaluated protein levels by IHC in FFPE tissues, whereas RNA-seq data were obtained from the TCGA. Therefore, it cannot be excluded that the inconsistency between studies may be attributed to discrepancies in the methods used. On the other hand, higher levels of mRNA *HNRNPU* were observed in tumoral vs. adjacent tissue in breast [11], hepatocellular [12], and bladder cancers [13]. Although there were no significant associations between the investigated clinicopathological features and HNRNPU protein expression levels, the survival analyses showed an adverse correlation between a high HNRNPU protein level and clinical outcomes of NSCLC patients. This observation was consistent with those made in previous studies that found the association between high HNRNPU and poor survival in patients with hepatocellular [12, 14], bladder [13], and neuroblastoma cancers [32]. In our study, HNRNPU protein (not mRNA expression) was an independent prognostic factor for predicting the survival of NSCLC patients. Regarding the TCGA dataset analyses, our results showed a trend, however, the outcomes did not reach statistical significance. Nevertheless, it should be emphasized that protein expression and mRNA levels are not necessarily always correlated. Based on data from The Gene Expression Omnibus dataset Li et al. showed that LSCC patients with high HNRNPU expression presented a notably longer OS and progression-free survival than those with low HNRNPU expression [31]. However, it may be observed that the direct comparison of mRNA expression data between our study and Li's research is limited by the fact that they come from different(RNA-seq. microarray) platforms, whose results are not necessarily compatible. Given the cited reports related to NSCLC, HNRNPU may be expected to act as a tumor suppressor; however, there is also evidence that supports its pro-tumor effect in different tumor types. Zhou et al. demonstrated that HNRNPU knockdown significantly inhibited the proliferation, migration, and invasion of BRCA cells in vitro [11]. Evidence indicates that HNRNPU knockdown in hepatocellular carcinoma cells inhibits cell growth and decreases chromatin accessibility [12]. Undoubtedly, these findings along with our results prove that HNRNPU plays an important role in carcinogenesis, and may serve as a novel prognostic biomarker.

In the final analyses, the possible effect of combined expression of RUVBL1 and HNRNPU on OS of NSCLC patients was investigated. The Kaplan–Meier survival curves revealed that OS was significantly worse in patients with co-expressed high protein levels of RUVBL1 and HNRNPU. Additionally, the combined expression of these markers proved to be a better predictor of patient survival than each of the factors independently. Our findings confirmed that the co-expression of high RUVBL1 and HNRNPU protein levels was a significant poor prognostic factor for OS with a particularly high hazard ratio as compared to each marker considered as a single indicator. We demonstrated similar relationships by analysing the mRNA of the markers studied from the TCGA cohort. Stratification of NSCLCs with respect to the combined expression of *RUVBL1* and *HNRNPU* mRNA allowed us to identify subgroups of patients with the largest survival differences. Furthermore, the combined mRNA panel was found to be a powerful independent prognostic factor correlated with poor outcome.

The major limitation of the study concerns small number of the samples for analyses at the protein level; however, it was still enough to demonstrate statistically strong effects, most of which are in agreement with both those obtained from the TCGA and those reported in the literature. It should be emphasized that the presented considerations integrate results obtained in slightly different patients’ populations; however still this is the most comprehensive report in the field, so far. Our observations require large-scale, and multicentre clinical studies to be confirmed. Also, there is a further need for description of biological background that could substantiate the presented observations. In the next studies we plan to perform genetic analyses of genes routinely evaluated in NSCLC (being diagnosed at early stage disease, the tumors from our cohort were not examined for *EGFR* and *KRAS* mutations, as well as *ALK* rearrangement), as well as target genes regulated by RUVBL1 and HNRNPU at the protein and mRNA levels.

To sum up, our findings provide solid and consistent associations of RUVBL1 and HNRNPU expression with NSCLC pathological and clinical course, that are compatible with the findings presented in previous studies. The results offer novel insights into the importance of RUVBL1 and HNRNPU and shed light on the possible application of these factors as biomarkers or even antineoplastic targets in NSCLC. Undoubtedly, further investigations are required to clarify the molecular mechanisms of the RUVBL1 and HNRNPU overexpression in NSCLC.

## Supplementary Information

Additional file1 (PDF 115 KB)

Additional file2 (DOCX 24 KB)

## Data Availability

The datasets generated during and/or analysed during the current study are available from the corresponding author on reasonable request.
