# Supplementary material for: High expression of RUVBL1 and HNRNPU is associated with poor overall survival in stage I and II non-small cell lung cancer patients
Source: Discov Oncol. 2022 Oct 15;13:106. doi: 10.1007/s12672-022-00568-0 (PMC9569266; doi:10.1007/s12672-022-00568-0)
Supplement: Supplementary file 1 — Additional file1 (PDF 115 KB) [file 12672_2022_568_MOESM1_ESM.pdf]

Supplementary Figure

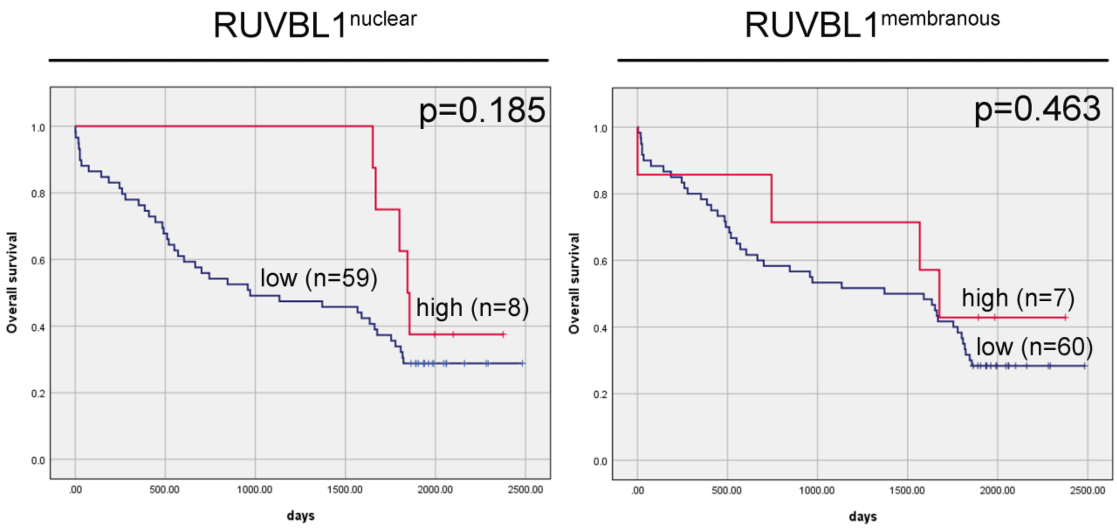

Supplementary Fig. 1 Kaplan-Meier survival curves presenting the prognostic impact of nuclear and membranous RUVBL expression levels detected by immunohistochemistry on OS of patients with NSCLC
