# Supplementary material for: High expression of RUVBL1 and HNRNPU is associated with poor overall survival in stage I and II non-small cell lung cancer patients
Source: Discov Oncol. 2022 Oct 15;13:106. doi: 10.1007/s12672-022-00568-0 (PMC9569266; doi:10.1007/s12672-022-00568-0)
Supplement: Supplementary file 2 — Additional file2 (DOCX 24 KB) [file 12672_2022_568_MOESM2_ESM.docx]

**Supplementary Tables**

**Supplementary Table 1** Clinicopathological characteristics of 67 patients with NSCLC from our cohort. ADC- adenocarcinoma; SCC - squamous cell carcinoma; LCC - large cell carcinoma; pT status – extent of the primary tumor; pN status – absence or presence and extent of regional lymph node metastasis

| Variables | Number (%) or  median ( 95.0% CI) |
| --- | --- |
| Histologic type |  |
| ADC | 26 (38.81) |
| SCC | 36 (53.73) |
| LCC | 5 (7.46) |
| Gender |  |
| Females | 21 (31.34) |
| Males | 46 (68.66) |
| Age |  |
| ≤62 | 30 (44.78) |
| >62 | 37 (55.22) |
| Smoking |  |
| Never | 3 (4.48) |
| Former/current | 64 (95.52) |
| Histologic grade |  |
| G2 | 19 (28.36) |
| G3 | 48 (71.64) |
| pT status |  |
| T1 | 25 (37.31) |
| T2 | 25 (37.31) |
| T3 | 17 (25.37) |
| pN status |  |
| N0 | 61 (91.04) |
| N1 | 6 (8.96) |
| TNM stage |  |
| IA | 22 (32.84) |
| IB | 14 (20.90) |
| IIA | 8 (11.94) |
| IIB | 23 (34.33) |
| Follow-up duration (days) | 1990 (1965-2014) |

**Supplementary Table 2** Clinicopathological properties of 761 patients with NSCLC from the TCGA cohort. pT status – extent of the primary tumor; pN status – absence or presence and extent of regional lymph node metastasis.

| Variables | Number (%) or  median ( 95.0% CI) |
| --- | --- |
| Gender |  |
| Females | 307 (40.34) |
| Males | 454 (59.66) |
| Age |  |
| <68 | 374 (49.15) |
| ≥68 | 387(50.85) |
| pT status |  |
| T1 | 249 (32.72) |
| T2 | 446 (58.61) |
| T3 | 66 (8.67) |
| pN status |  |
| N0 | 590 (77.53) |
| N1 | 171 (22.47) |
| TNM stage |  |
| I | 488 (64.13) |
| II | 273 (35.87) |
| Follow-up duration (days) | 913 (826-1000) |

**Supplementary Table 3** Comparison of the clinicopathological properties of our own cohort and the TCGA cohort. pT status – extent of the primary tumor; pN status – absence or presence and extent of regional lymph node metastasis.

| **Variables** | **Our cohort** | **TCGA cohort** | **P-value** |
| --- | --- | --- | --- |
|  |  |  |  |
| **Gender** |  |  |  |
| **Females** | 21 (31.34) | 307 (40.34) | 0.1546 |
| **Males** | 46 (68.66) | 454 (59.66) |  |
| **Age** |  |  |  |
| **<67** | 42 (62.69) | 349 (45.86) | **0.0103** |
| **≥67** | 25 (37.31) | 412 (54.14) |  |
| **pT status** |  |  |  |
| **T1** | 25 (37.31) | 249 (32.72) | **<0.0001** |
| **T2** | 25 (37.31) | 446 (58.61) |  |
| **T3** | 17 (25.37) | 66 (8.67) |  |
| **pN status** |  |  |  |
| **N0** | 61 (91.04) | 590 (77.53) | **0.0078** |
| **N1** | 6 (8.96) | 171 (22.47) |  |
| **TNM stage** |  |  |  |
| **I** | 36 (53.73) | 488 (64.13) | 0.112 |
| **II** | 31 (46.27) | 273 (35.87) |  |

**Supplementary Table 4** Association of nuclear and membranous RUVBL1 and clinicopathological features in our cohort of NSCLC patients. ADC- adenocarcinoma; SCC - squamous cell carcinoma; LCC - large cell carcinoma; pT status – extent of the primary tumor; pN status –absence or presence and extent of regional lymph node metastasis.

|  |  | **RUVBL1^nucleus^** | | | **RUVBL1^membranous^** | | |
| --- | --- | --- | --- | --- | --- | --- | --- |
|  | Cases | + | - | P-value | + | - | P-value |
|  | n (%) | n = 8 | n = 59 |  | n = 7 | n = 60 |  |
| **Histological type** | |  |  |  |  |  |  |
| **ADC** | 26 (38.81) | 2 (7.69) | 24 (92.31) | 0,7515 | 3 (11.54) | 23 (88.46) | 0,5939 |
| **SCC** | 36 (53.73) | 6 (16.67) | 30 (83.33) |  | 4 (11.11) | 32 (88.89) |  |
| **LCC** | 5 (7.46) | 0 (0.00) | 5 (100.00) |  | 0 (0.00) | 5 (100.00) |  |
| **Gender** |  |  |  |  |  |  |  |
| **Females** | 21 (31.34) | 2 (9.52) | 19 (90.48) | >0,9999 | 2 (9.52) | 19 (90.48) | >0,9999 |
| **Males** | 46 (68.66) | 6 (13.04) | 40 (86.96) |  | 5 (10.87) | 41 (89.13) |  |
| **Age** |  |  |  |  |  |  |  |
| **<62** | 30 (44.78) | 5 (16.67) | 25 (83.33) | 0,4512 | 3 (10.00) | 27 (90.00) | >0,9999 |
| **≥62** | 37 (55.22) | 3 (8.11) | 34 (91.89) |  | 4 (10.81) | 33 (89.19) |  |
| **Histologic grade** | |  |  |  |  |  |  |
| **G2** | 19 (28.36) | 2 (10.53) | 17 (89.47) | >0,9999 | 2 (10.53) | 17 (89.47) | >0,9999 |
| **G3** | 48 (71.64) | 6 (12.50) | 42 (87.50) |  | 5 (10.42) | 43 (89.58) |  |
| **pT status** |  |  |  |  |  |  |  |
| **T1** | 25 (37.31) | 2 (8.00) | 23 (92.00) | 0,6833 | 4 (16.00) | 21 (84.00) | 0,1064 |
| **T2** | 25 (37.31) | 4 (16.00) | 21 (84.00) |  | 3 (12.00) | 22 (88.00) |  |
| **T3** | 17 (25.37) | 2 (11.76) | 15 (88.24) |  | 0 (0.00) | 17 (100.00) |  |
| **pN status** |  |  |  |  |  |  |  |
| **N0** | 61 (91.04) | 8 (13.11) | 53 (86.89) | >0,9999 | 7 (11.48) | 54 (88.52) | >0,9999 |
| **N1** | 6 (8.96) | 0 (0.00) | 6 (100.00) |  | 0 (0.00) | 6 (100.00) |  |
| **Stage** |  |  |  |  |  |  |  |
| **I** | 36 (53.73) | 5 (13.89) | 31 (86.11) | 0,7158 | 3 (8.33) | 29 (80.56) | >0,9999 |
| **II** | 31 (46.27) | 3 (9.68) | 28 (90.32) |  | 4 (12.90) | 27 (87.10) |  |
